# Repetitive transcranial magnetic stimulation treatment of major depressive disorder and comorbid chronic pain: response rates and neurophysiologic biomarkers

**DOI:** 10.1017/S0033291721002178

**Published:** 2023-02

**Authors:** Juliana Corlier, Reza Tadayonnejad, Andrew C Wilson, Jonathan C Lee, Katharine G Marder, Nathaniel D Ginder, Scott A Wilke, Jennifer Levitt, David Krantz, Andrew F Leuchter

**Affiliations:** 1 TMS Clinical and Research Service, Neuromodulation Division, Semel Institute for Neuroscience and Human Behavior, and the Department of Psychiatry and Biobehavioral Sciences, David Geffen School of Medicine at UCLA, 760 Westwood Plaza, Los Angeles, CA 90024, USA; 2 Division of the Humanities and Social Sciences, California Institute of Technology, Pasadena, CA 91125, USA; 3 VA Greater Los Angeles Healthcare System, 11301 Wilshire Blvd, Los Angeles, CA 90073, USA

**Keywords:** Major depressive disorder (MDD), Chronic Pain, Repetitive transcranial magnetic stimulation (rTMS), peak alpha frequency (PAF), phase coherence

## Abstract

**Background:**

Major depressive disorder (MDD) and chronic pain are highly comorbid, and pain symptoms are associated with a poorer response to antidepressant medication treatment. It is unclear whether comorbid pain also is associated with a poorer response to treatment with repetitive transcranial magnetic stimulation (rTMS).

**Methods:**

162 MDD subjects received 30 sessions of 10 Hz rTMS treatment administered to the left dorsolateral prefrontal cortex (DLPFC) with depression and pain symptoms measured before and after treatment. For a subset of 96 patients, a resting-state electroencephalogram (EEG) was recorded at baseline. Clinical outcome was compared between subjects with and without comorbid pain, and the relationships among outcome, pain severity, individual peak alpha frequency (PAF), and PAF phase-coherence in the EEG were examined.

**Results:**

64.8% of all subjects reported pain, and both depressive and pain symptoms were significantly reduced after rTMS treatment, irrespective of age or gender. Patients with severe pain were 27% less likely to respond to MDD treatment than pain-free individuals. PAF was positively associated with pain severity. PAF phase-coherence in the somatosensory and default mode networks was significantly lower for MDD subjects with pain who failed to respond to MDD treatment.

**Conclusions:**

Pain symptoms improved after rTMS to left DLPFC in MDD irrespective of age or gender, although the presence of chronic pain symptoms reduced the likelihood of treatment response. Individual PAF and baseline phase-coherence in the sensorimotor and midline regions may represent predictors of rTMS treatment outcome in comorbid pain and MDD.

## Introduction

Major depressive disorder (MDD) and chronic pain are highly comorbid, especially in female patients (Bair et al., 2004). This comorbidity significantly decreases the quality of life of patients and represents one of the largest socioeconomic burdens worldwide (Leuchter et al., 2010; Walker, Kavelaars, Heijnen, & Dantzer, 2014). Pain symptoms can either precede or follow the onset of MDD (Bair, Robinson, Katon, & Kroenke, 2003; Chang et al., 2015; Gerrits, Van Oppen, Van Marwijk, Penninx, & Van Der Horst, 2014), suggesting a bidirectional relationship between MDD and chronic pain. Several studies have suggested that affective disorders and chronic pain have an overlapping pathophysiology and may share similar circuit mechanisms (Bair et al., 2003; Taylor, Becker, Schweinhardt, & Cahill, 2016). While both depression and pain symptoms can be alleviated by antidepressant medications (Gracely, Ceko, & Bushnell, 2012; Maletic & Raison, 2009), this comorbidity has generally been associated with greater resistance to pharmacological treatment (Bair et al., 2004, 2003; Gerrits et al., 2012; Leuchter et al., 2010; Von Korff & Simon, 1996). In particular, pain severity has been reported to be a strong predictor of poorer antidepressant medication treatment outcome and health-related quality of life (Bair et al., 2004). Better recognition, assessment, and treatment of comorbid pain may thus enhance the outcome of antidepressant therapy.

Repetitive transcranial magnetic stimulation (rTMS) administered to the left dorsolateral prefrontal cortex (DLPFC) is an effective treatment for pharmaco-resistant depression (George et al., 2010; Janicak et al., 2013). While the mechanism of action (MOA) of this neuromodulation technique is not yet fully understood, there is evidence to suggest that the therapeutic effect of rTMS arises through the resetting of resting-state functional networks beyond the stimulation site (Corlier et al., 2019*a*, *b*; Fox, Halko, Eldaief, & Pascual-Leone, 2012; Leuchter, Hunter, Krantz, & Cook, 2015; To, De Ridder, Hart, & Vanneste, 2018). rTMS also appears to be efficacious for the treatment of other neuropsychiatric disorders including post-traumatic stress disorder, obsessive-compulsive disorder (Tadayonnejad et al., 2020), generalized anxiety disorder, bipolar depression, tinnitus, neurodegenerative disorders (Carpenter et al., 2018; Heath, Taylor, & McNerney, 2018; Lefaucheur et al., 2014, 2020; Soleimani, Jalali, & Hasandokht, 2016), and pain syndromes such as neuropathic pain, headache, fibromyalgia, and complex regional pain syndrome (Altas, Askin, Beşiroğlu, & Tosun, 2019; Galhardoni et al., 2015; Goudra et al., 2017; Hou, Wang, & Kang, 2016; Hsu, Daskalakis, & Blumberger, 2018; Knijnik et al., 2016; Saltychev & Laimi, 2017; Short et al., 2011). Most studies of rTMS on pain have targeted the primary motor cortex, but stimulation to left DLPFC has also successfully reduced pain symptoms even in non-MDD populations (Galhardoni et al., 2015; Johnson, Summers, & Pridmore, 2006; Lefaucheur et al., 2014; Short et al., 2011). One previous report demonstrated significant improvement in both mood and pain symptoms solely with 10 Hz rTMS treatment applied to left DLPFC (Phillips, Burr, & Dunner, 2018). However, it remains unclear whether this effect was gender-specific and whether the presence and severity of pain symptoms was associated with inferior rTMS clinical outcome, as is the case with pharmacological treatment.

Identifying neurophysiological biomarkers of chronic pain would aid in the development of an rTMS protocol targeting this comorbidity. Such measures would allow the assessment of target engagement and could serve as early predictors of treatment outcome. Alpha band oscillations have previously been identified as a robust electroencephalographic (EEG) marker of chronic pain and might represent a possible biomarker for rTMS. For example, higher alpha power has been observed within the dynamic pain connectome in subjects with neuropathic pain, rheumatoid arthritis, and jaw pain (Kim & Davis, 2020; Kisler et al., 2020; Meneses et al., 2016; Wang et al., 2019), while decreased alpha oscillations have been reported in tonic pain (cf. Ploner, Sorg, & Gross, 2017). Lower peak alpha frequency [PAF, also called individual alpha frequency (IAF)] (Grandy et al., 2013; Petrosino, Zandvakili, Carpenter, & Philip, 2018) also has been observed in neuropathic pain and fibromyalgia (Kim et al., 2019). Sensorimotor PAF has been reported as a reliable biomarker of subjective pain intensity and pain sensitivity, with slower PAF possibly reflecting pre-disease pain sensitivity (Babiloni et al., 2006; Furman et al., 2018, 2019, 2020) or marking a ‘chronification’ process of pain (de Vries et al., 2013). Targeted modulation of alpha activity through visual stimulation, as well as transcranial alternating or direct current stimulation, also has been associated with chronic pain relief (Ahn, Prim, Alexander, McCulloch, & Fröhlich, 2019; Arendsen, Hugh-Jones, & Lloyd, 2018; Lopez-Diaz et al., 2021). Recent evidence indicates that alpha synchrony between relevant networks enables feedforward/feedback processing and flexible routing of information in the integration of sensory and contextual processes (Kim & Davis, 2020; Kisler et al., 2020; Ploner et al., 2017). The examination of PAF/IAF and synchrony in the nociceptive network may therefore serve as an effective biomarker of target engagement and clinical response for rTMS treatment of chronic pain and depression.

Given the prevalence of comorbid chronic pain and MDD, and in light of their overlapping pathophysiology, it is of crucial importance to elucidate novel targets and integrated interventions to treat comorbid pain and depression, rather than targeting pain and depressive symptoms separately (Walker et al., 2014). The primary objectives of this study were thus to: (1) evaluate the effect of pain comorbidity on rTMS treatment response for MDD; (2) examine gender differences in rTMS treatment outcome; and (3) examine IAF and alpha band network synchrony as potential biomarkers for rTMS treatment outcome for comorbid MDD and chronic pain.

## Methods

### Subjects

**Fig. 1.**
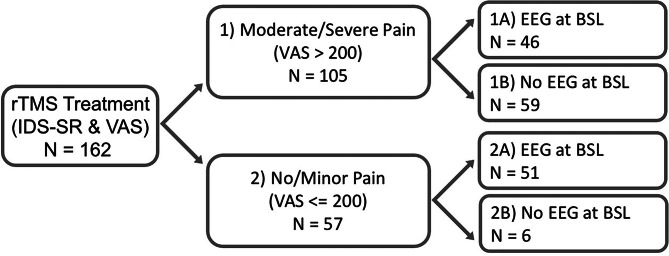
Breakdown of total sample into subgroups. The total sample of *N* = 162 was split into Groups 1 and 2 based on the presence of pain symptoms (cutoff: VAS>200). Each subgroup was further divided depending on whether subjects had a baseline EEG recording (Groups 1A and 2A) or not (Groups 1B and 2B).

Subjects were 162 outpatients with a primary diagnosis of MDD (Mini International Diagnostic Interview, MINI) (Sheehan et al., 1998) referred for treatment in the TMS Clinical and Research Service at UCLA. The research protocol was approved by the UCLA IRB and all subjects provided informed consent prior to research procedures. Subjects presented with at least moderately severe depressive symptoms based upon a 17-item Hamilton Depression Rating Scale Score (HAM-D17, minimal score >17) (Hamilton, 1960) and had failed to enter remission after at least three adequate antidepressant trials. Subjects were allowed to continue receiving psychotropic medication concurrent with rTMS and underwent standard safety screening and medical clearance before receiving rTMS treatment. The entire sample of 162 MDD subjects who completed a 30-session rTMS course for depression was categorized into subgroups as follows: Group (1) 105/162 who reported pretreatment comorbid pain; Group (2) 57/162 with no comorbid pain; Group (1A) 46/105 comorbid subjects with a spontaneous EEG recorded prior to the first session; Group (1B) 59/105 without EEG; Group (2A) 51/57 pain-free subjects with a baseline EEG recording; Group (2B) 6/57 pain-free subjects without EEG (see also Fig. 1). All procedures in this work comply with the ethical standards of the relevant national and institutional committees on human experimentation and with the Helsinki Declaration of 1975 as revised in 2008.

### Clinical assessment

Depressive symptoms were assessed at baseline and session 30 with the 30-item Inventory of Depressive Symptomatology Self Report version (IDS-SR) (Trivedi et al., 2004). The reliability of IDS-SR is demonstrated by its use as the primary outcome in STAR*D, the largest open-label, pragmatic trial for MDD (Sinyor, Schaffer, & Levitt, 2010). Chronic pain was assessed at baseline and treatment 30 using the eight-question Visual Analog Scale (VAS) for pain with total scores ranging from 0 to 800 (Chiarotto et al., 2019). Each question ranges [0–100] and assesses, during the last week, overall pain, along with separate items for headache, back, shoulder, stomach, and abdominal pain, interference with daily function, and nighttime awakenings due to pain. The VAS has been widely used in adult patients with various chronic pain conditions (Mori et al., 2010; Onesti et al., 2013) and has been established as a reliable measure in a review of over 850 studies (Karcioglu, Topacoglu, Dikme, & Dikme, 2018). Patients with different pain types were included in this sample, including fibromyalgia-like symptoms, neuropathic pain, chronic lower back pain, and complex regional pain syndrome, and the specific source of chronic pain for each individual was not formally diagnosed in the context of this study. We therefore were not able to perform separate analyses for each pain type.

### EEG acquisition

Ninety-seven of 162 subjects had an EEG recording performed at baseline using the ANT Neuro TMS-compatible EEG system and EEGO v1.8 recording software (Advanced Neuro Technology (ANT); Enschede, the Netherlands). Electrodes were applied using the 64-electrode ‘WaveGuard’ cap with sintered Ag/AgCl electrodes positioned according to the Extended 10–20 System with EOG electrodes above and below the left eye. Data were recorded at a sampling rate of 2000 Hz using full-band EEG DC amplifiers without filters during data acquisition using a CPz electrode reference; data were converted to a common average reference offline for analysis. Impedances were kept below 10 kΩ. All subjects had at least 5 min of baseline resting-state EEG recording.

### rTMS procedures

All TMS treatments were performed with either the Magstim Rapid 2 stimulator using a 70 mm coil (Magstim, Whitland, South Wales, UK), the Neuronetics Neurostar treatment system (Neuronetics, Malvern, PA, USA), or MagVenture (Alpharetta, GA, USA). Motor threshold (MT) determination was performed prior to the first treatment, with MT defined as the minimum stimulus intensity necessary to elicit an overt motor response in the right abductor pollicis brevis or first dorsal interosseus muscles for ⩾50% of applied stimuli. Following MT determination, treatments were performed with patients seated in a semi-reclined position using standard safety procedures and ear protection. All patients underwent treatment initially with 10 Hz stimulation to left DLPFC defined using the Beam F3 method (Beam, Borckardt, Reeves, & George, 2009), with a 40-pulse train and 26 s intertrain interval with a total of 3000 pulses per session (37.5 min duration). Clinicians adjusted stimulation intensity, coil angle, and number of pulses administered as needed to optimize tolerability. Treatment intensity was titrated to 120% MT as tolerated with parameters modified according to a measurement-based care paradigm. Participants completed IDS-SR ratings every five treatments. If there was an absence of early clinical response to rTMS or worsening in anxiety or depressive symptoms, the treatment protocol could be complemented by intermittent theta-burst stimulation priming (iTBS) at left DLPFC (Lee et al., 2020) or by 1 Hz rTMS at the right DLPFC after the fifth session to optimize tolerability or augment clinical response (sequential bilateral treatment). In the total group of 162 subjects, 137 (84.5%) received modified treatment parameters and the use of these parameters was included as a categorical covariate in the analysis of variance.

### Data analysis

**Fig. 2.**
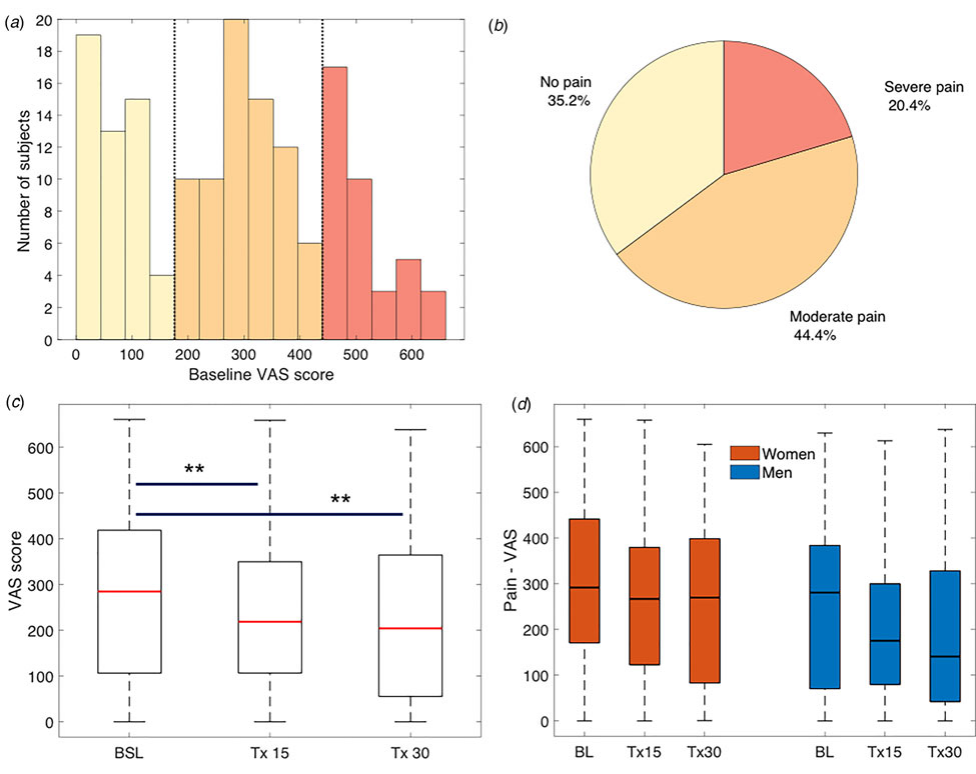
Pain prevalence and change with rTMS treatment. (*a*) The reported pain severity followed a trimodal distribution, corresponding to three empirically defined groups with ‘No Pain’, ‘Moderate pain’, or ‘Severe pain’. (*b*) Most MDD patients (64.8%) presented with comorbid pain. (*c*) Pain symptoms significantly decreased with rTMS treatment for depression after 15 and 30 sessions. (*d*) There were no gender differences in reported pain severity or improvement.

We performed a *t* test comparing pre- to post-rTMS IDS-SR scores of the entire sample to assess the therapeutic efficacy of rTMS treatment. To characterize pain comorbidity in the current sample, we calculated proportions of MDD patients with no, moderate, or severe pain using the VAS scale for pain based on the observed trimodal distribution of the pain scores (Fig. 2*a*). Following the trimodal distribution of these data, we grouped subjects into (1) no pain or no significant chronic pain (termed ‘no pain’ or ‘pain-free’ subjects), (2) moderate pain, or (3) severe pain. The cutoffs were as follows: no pain: VAS = [0–200]; moderate pain: VAS = [201–450]; and severe pain: VAS = [451–800]. The proportions of subjects in each category were calculated.

We then calculated Pearson's correlation coefficients to evaluate the link (1) between baseline depression and pain severity, (2) between final outcome in depressive and pain symptoms, as well as (3) between baseline pain and final depression outcomes. Because pain and somatic symptom ratings were included in the overall IDS score, we also recalculated the IDS after removing pain- and sleep-related items [1 to 4 (sleep-related) and 20, 25, 26, 30 (pain- and energy-related)], to test whether relationships between depression and pain were affected by the removal of pain and somatic items [this reduced measure referred to as IDS-SR(22)].

An analysis of variance (ANOVA) was conducted to examine the effect of rTMS treatment (factor Treatment #) on pain including the five following covariates: age, gender, TMS device (1–3), use of modified rTMS protocol (yes/no), concomitant use of psychotropic medication during rTMS treatment (yes/no). Additionally, we compared response and remission rates for depression among groups of varying pain levels with response defined as a ⩾50% decrease in IDS-SR score from baseline and remission was defined as an IDS-SR score of ⩽13. Clinical rTMS response and remission rates across the three groups were compared using a Kruskal-Wallis test.

### EEG analyses

EEGs (conducted on Groups 1A and 2A) were preprocessed using the semi-automated FASTER toolbox (Nolan, Whelan, & Reilly, 2010), an ICA-based algorithm to remove eye movement, muscular, and line noise artifacts, followed by visual inspection to manually remove remaining artifacts. Individual PAF was estimated using two complementary approaches: (1) a ‘center of gravity’ (COG) method previously used in pain research by Furman and colleagues (Seminowicz et al., 2018), and (2) the IAF method used in MDD research by Corlier et al. (2019*a*, *b*). In both cases, the PAF was estimated based on the averaged power spectral density of postcentral channels Cz, C3, and C4 using a common average reference. We evaluated the relationship between IAF and pain severity by computing the Pearson's correlation coefficient between COG/IAF and the VAS baseline scores only for the patients with MDD and pain comorbidity (i.e. with a minimal VAS score >200).

**Fig. 3.**
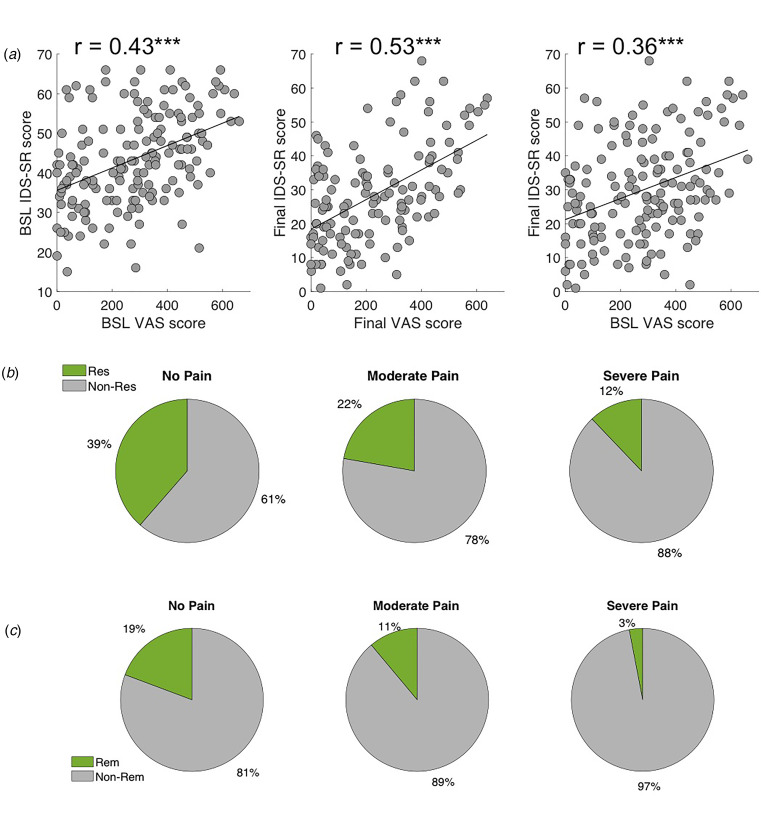
Association between depressive and pain symptoms and clinical outcome. (*a*) Left: Baseline pain severity was significantly associated with baseline depression severity. Center: Final pain outcome was significantly associated with final depression outcome; and Right: Baseline pain severity was significantly associated with final depression outcome. (*b*) Clinical response rates for different pain groups. Probability of response to rTMS was smallest for the severe pain group. (*c*) Clinical remission rates for different pain groups. Probability of remission with rTMS was smallest for the severe pain group.

We then performed phase-coherence analysis using channels-of-interest (ChOI) corresponding to the surface areas above motor, somatosensory, anterior and posterior cingulate cortices, as well as the precuneus. The 14 ChOI included: Fz, FCz, Cz, CPz, Pz, POz, FC1, FC2, FC3, FC4, C1, C2, C3, and C4 (ChOI are marked by blue spheres in Fig. 3*c*/*d*) with 91 total possible pairwise connections. Phase-coherence was calculated using the weighted phase lag index (wPLI), which has the advantage of yielding a reliable estimate even in noisy conditions. This metric weighs the observed phase leads and lags by the magnitude of the imaginary component of the cross-spectrum, is less sensitive to volume conduction, and provides greater statistical power to detect changes in phase-synchronization (Vinck, Oostenveld, Van Wingerden, Battaglia, & Pennartz, 2011; Xing et al., 2016a, 2016b). WPLI was estimated based on 4 s segments of artifact-free EEG data using the fieldtip toolbox function ‘ft_connectivityanalysis’. Baseline levels of wPLI were compared between comorbid responders/remitters and non-responders/non-remitters using *t* tests. Benjamini-Hochberg False Discovery Rate (FDR) method was used for the correction of multiple comparisons. We chose the top 25 connections with the largest Cohen's *D* effect sizes to compare the average network connectivity between responders/remitters and non-responders/non-remitters for the comorbid and the pain-free groups.

## Results

In total, 35.2% of MDD subjects presented with no pain, 44.4% of subjects reported moderate pain, and 20.4% had severe pain symptoms, exhibiting a trimodal distribution of severity (Fig. 2*a* and *b*). Baseline and post-treatment depression scores were significantly higher in the comorbid than in the pain-free MDD individuals. There was no effect of age or gender on baseline pain severity (Table 1). On average, rTMS treatment significantly reduced depressive (two-tailed *t* test, *T* = 14.0, *p* < 0.0001) and pain symptoms (ANOVA, *F* = 3.8, *p* = 0.02), and posthoc *t* tests showed pain improvement by treatment 15 with further improvement seen at treatment 30 (*T*_15_, *p*_15_ = 0.0088, *T*_30_, *p*_30_ = 0.0067, Fig. 1*c*). There was no significant effect of gender (*F* = 2.4, *p* = 0.09, Fig. 1*d*), age (*F* = 0.1, *p* = 0.7), TMS device (*F* = 0.1, *p* = 0.9), use of psychotropic medication (*F* = 0.7, *p* = 0.4), or use of modified rTMS protocol (*F* = 2.7, *p* = 0.1) on outcome (Table 1).

**Table 1.** Demographic and clinical information for all subjects and those with and without pain

	All subjects *N* *=* 162	MDD + no pain (VAS ≤ 200) *N* *=* 57	MDD + pain (VAS > 200) *N* *=* 105	Test statistic	*p* value
Age (years)	49.4 ± 16.5	45.3 ± 14.5	44.6 ± 15.9	*T* = −0.17	*p* = 0.87
Gender (% female)	*N* = 90 55.6%	*N* = 29 50.9%	*N* = 61 58.1%	χ^2^ = 0.78	*p* = 0.38
Psychotropic medication	*N* = 141 87%	*N* = 50 87.7%	*N* = 91 86.7%	*F* = 0.7	*p* = 0.4
Baseline IDS-SR	43.4 ± 11.7	38.5 ± 11.7	46.0 ± 10.8	*T* = −4.1	*p* < 0.001
Tx 15 IDS-SR	33.7 ± 13.5	29.1 ± 12.8	36.3 ± 13.2		
Final IDS-SR	29.8 ± 14.9	24.7 ± 14.0	32.6 ± 14.7	*T* = −3.2	*p* = 0.0016
Baseline VAS	276.7 ± 175.5	79.5 ± 58.8	383.8 ± 113.8	*T* = 18.9	*p* < 0.001
Tx 15 VAS	246.1 ± 160.8	119.3 ± 89.3	313.1 ± 149.6		
Final VAS	238.1 ± 180.2	99.7 ± 131.3	312.9 ± 157.8	*T* = 7.3	*p* < 0.001

There was no age or gender difference between groups. Both initial depression severity and final depression score were significantly higher in the pain group.

There was a significant positive correlation between the severity of pain and depression at baseline (*r* = 0.43, *p* = 1.56 × 10^−08^), between the final pain and depression outcome (*r* = 0.53, *p* = 9.59 × 10^−11^), and between baseline pain severity and final depression outcome (*r* = 0.36, *p* = 3.73 × 10^−06^). Response rates were significantly different across the three pain severity groups (χ^2^ = 8.5, *p* = 0.014), with 27% lower rTMS response probability among patients with more severe pain compared to the pain-free group (rTMS response rates of 39, 22, and 12% rates for no, moderate, and severe pain, respectively, Fig. 2*c*). Differences in remission rates among the three groups reached trend-level significance (χ^2^ = 5.26, *p* = 0.072, rTMS remission rates of 19, 11, and 3% for no, moderate, or severe pain, respectively). The significant association between pain and depression symptoms persisted even after removing eight pain, sleep, and energy-related items from the IDS-SR (*r* = 0.32, *p* = 0.008 at baseline and *r* = 0.35, *p* = 0.003 at endpoint).

**Fig. 4.**
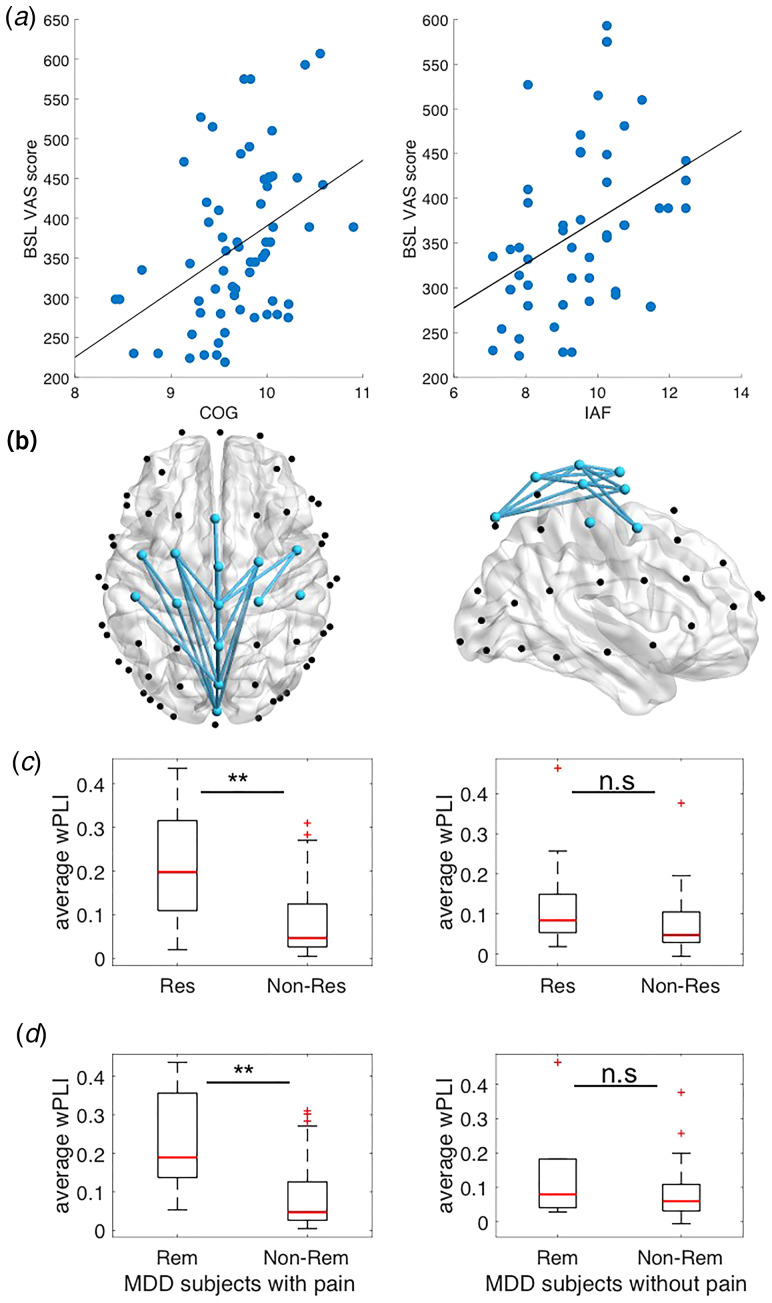
Association between pain symptoms, rTMS response and EEG measures for comorbid subjects (VAS score>200). (*a*) Left: Higher COG was associated with higher baseline VAS score. Right: Higher IAF was associated with higher baseline VAS score. (*b*) Left: Phase-coherence was reduced at sensorimotor-midline locations for comorbid non-responders as compared to responders (axial and sagittal views); Right: There was no such difference between pain-free responders and non-responders. (*c*) Left: The average phase-coherence of all connections was significantly reduced for comorbid non-responders compared to responders; Right: There was no such difference between pain-free responders and non-responders.(*d*) Left: The average phase-coherene of all connections was significantly reduced for comorbid non-remitters compared to remitters; Right: There was no such difference between pain-free remitters and non-remitters.

EEG data in the comorbid patient subgroup revealed that a higher individual PAF was associated with more severe chronic pain complaints at baseline. This was confirmed for both COG and PAF measures (COG: *r* = 0.41, *p* = 0.00095; PAF: *r* = 0.39, *p* = 0.0047, Fig. 3*a* and *b*). Phase-coherence for the top 25 connections was significantly higher for comorbid responders/remitters than for non-responders/non-remitters at a level of *p* < 0.05 after FDR correction (Fig. 4*b*, Table 2). The average network connectivity across these 25 connections was also overall significantly different between responders/non-responders (*t* = 3.4, *p* = 0.001) and remitters/non-remitters (*t* = 3.1, *p* = 0.003; Fig. 4*c* and *d*, left panel). However, in the same network, there was no difference between pain-free responders/remitters and non-responders/non-remitters (*t* = 1.7, *p* = 0.1 and *t* = 1.3; *p* = 0.2, respectively; Fig. 4*c* and *d*, right panel).

**Table 2.** Listing of top 25 electrode pairs with largest Cohen's *D* effect sizes for differences in baseline phase-coherence between comorbid responders and non-responders to rTMS treatment

	Chan 1	Chan 2	wPLI responders mean ± s.d.	wPLI non-responders mean ± s.d.	*T* value	Raw *p* value	Adj *p* value	Cohen's *D*
1	Fp1	PO3	0.21 ± 0.12	0.08 ± 0.09	3.4503	0.0012	0.0178	1.2282
2	Fp1	O1	0.16 ± 0.1	0.06 ± 0.07	3.4788	0.0011	0.0178	1.1935
3	Fp1	F3	0.21 ± 0.12	0.09 ± 0.1	3.2669	0.0021	0.0178	1.1638
4	Fp1	P1	0.28 ± 0.2	0.09 ± 0.12	3.5926	8.21 × 10^−04^	0.0178	1.1444
5	Fpz	F8	0.26 ± 0.17	0.1 ± 0.11	3.4286	0.0013	0.0178	1.1388
6	Fpz	C3	0.25 ± 0.16	0.1 ± 0.1	3.4069	0.0014	0.0178	1.1272
7	Fp1	P2	0.27 ± 0.19	0.09 ± 0.11	3.5064	0.0011	0.0178	1.1125
8	Fp1	F4	0.24 ± 0.14	0.1 ± 0.11	3.121	0.0032	0.0178	1.1034
9	Fp1	P6	0.27 ± 0.19	0.1 ± 0.12	3.3508	0.0017	0.0178	1.0957
10	Fpz	CP1	0.25 ± 0.16	0.1 ± 0.11	3.1989	0.0026	0.0178	1.0804
11	Fp1	PO4	0.12 ± 0.08	0.05 ± 0.06	3.1996	0.0026	0.0178	1.0636
12	Fpz	AF7	0.15 ± 0.11	0.06 ± 0.08	3.0846	0.0035	0.0178	1.0512
13	Fp1	T8	0.17 ± 0.1	0.07 ± 0.09	2.7755	0.0081	0.0262	1.0506
14	Fp1	CP2	0.22 ± 0.15	0.09 ± 0.09	3.257	0.0022	0.0178	1.0502
15	Fp1	FC5	0.23 ± 0.14	0.1 ± 0.1	2.9836	0.0046	0.0211	1.0417
16	Fp1	CPz	0.24 ± 0.18	0.09 ± 0.12	2.9461	0.0051	0.0212	0.9875
17	Fp1	CP1	0.23 ± 0.17	0.1 ± 0.1	3.1402	0.003	0.0178	0.9841
18	Fpz	P4	0.14 ± 0.1	0.06 ± 0.06	3.0919	0.0034	0.0178	0.9741
19	Fpz	C4	0.21 ± 0.17	0.08 ± 0.09	3.1084	0.0033	0.0178	0.9531
20	Fp1	AF7	0.23 ± 0.17	0.09 ± 0.11	2.8768	0.0062	0.0235	0.9509
21	Fp1	CP6	0.22 ± 0.16	0.1 ± 0.1	2.8592	0.0065	0.0235	0.9444
22	Fp1	AF3	0.23 ± 0.16	0.09 ± 0.11	2.7969	0.0076	0.0257	0.9434
23	Fpz	T7	0.16 ± 0.12	0.07 ± 0.08	2.8622	0.0064	0.0235	0.9277
24	Fpz	Fz	0.23 ± 0.2	0.09 ± 0.1	3.0442	0.0039	0.0188	0.921
25	Fp1	FCz	0.13 ± 0.1	0.05 ± 0.04	3.2866	0.002	0.0178	0.9114

All connections were significantly different between the groups at the level of *p* < 0.05, FDR-corrected.

## Discussion

More than half of the MDD subjects in our sample presented with moderate to severe chronic pain symptoms, independent of age or gender, and the severity of depressive and pain symptoms were highly correlated. rTMS treatment was efficacious in reducing both comorbid symptoms although the presence of pain was associated with a worse antidepressant rTMS outcome. EEG analysis revealed that individual PAF was positively associated with chronic pain severity while baseline phase-coherence along the midline and sensorimotor regions was significantly lower among non-responders/non-remitters than in responders/remitters with comorbid pain.

Our finding that most MDD subjects report chronic pain symptoms that are associated with a poorer rTMS treatment outcome is consistent with the prior literature on medication treatment of MDD. The present findings confirm the high prevalence of this comorbidity and emphasize the need for a better understanding of the shared pathophysiology, as well as development of novel treatment strategies targeting both disorders (Bair et al., 2004). This study did not confirm previous reports of age- or gender-specific differences in pain prevalence and response (Phillips et al., 2018). Women and men in this study were equally likely to report and improve in pain regardless of age, although there was a trend-level effect for gender with females responding at a smaller degree. Failure to separate pain intensity and pain unpleasantness may underlie this discrepancy; future studies should attempt to more clearly distinguish these elements of pain.

Baseline depressive and pain symptoms were positively correlated, and better depression outcomes correlated with greater pain improvement, even after removing the somatic symptoms from the IDS-SR. It is thus difficult to dissociate the pain response from the antidepressant effect of the rTMS treatment. Further studies are necessary to characterize the interaction of separate or common brain circuits that may be involved. These data suggest, however, that rTMS to left DLPFC may be a less effective treatment for depressed patients with comorbid pain than those without pain. This finding is consistent with prior studies that have reported a poorer outcome for MDD-pain comorbid patients when treated with medications (Bair et al., 2003; Gerrits et al., 2012; Leuchter et al., 2010). It is also in line with previous findings showing that higher baseline depression severity is associated with poorer clinical response to rTMS (Fitzgerald, Hoy, Anderson, & Daskalakis, 2016; Janicak et al., 2013; Krepel, Rush, Iseger, Sack, & Arns, 2019). We hypothesize, however, that the distinct MOA of rTMS may be better suited to target the shared neurophysiological pathways of depression and comorbid pain than antidepressant medication.

The present results confirm that rTMS administered to left DLPFC does ameliorate comorbid pain symptoms in MDD. Combined with the broad prior treatment literature, however, these findings suggest that a multi-target rTMS approach for the combined treatment of MDD and chronic pain may therefore represent a promising alternative therapeutic approach. Future studies should systematically evaluate possible secondary targets for rTMS treatment of pain, such as the primary motor cortex, ventromedial prefrontal cortex, or the anterior cingulate cortex, which all have been linked to pain processing or pain relief after rTMS. Given that difficult-to-treat MDD patients with chronic pain are more likely to be administered opioids for pain relief, a novel rTMS therapeutic approach may help reduce opioid prescriptions and drug dependency for these challenging comorbid patients (Cahill & Taylor, 2017; Sullivan, Edlund, Steffick, & Unützer, 2005).

These results also extend previous findings of an association between individual PAF and pain susceptibility to acute and prolonged experimentally induced pain (Furman et al., 2018; Seminowicz et al., 2018) and in chronic pain conditions (Babiloni et al., 2006; de Vries et al., 2013; Furman et al., 2019, 2020). Individual PAF represents a stable individual trait (Grandy et al., 2013; Petrosino et al., 2018) that multiple studies have shown to be associated with rTMS treatment outcome in MDD (Arns, Drinkenburg, Fitzgerald, & Kenemans, 2012; Corlier et al., 2019*a*, *b*). While our observation stands in contrast to previously reported alpha-band slowing in multiple sclerosis subjects with neuropathic pain (Kim et al., 2019), this discrepancy may be due to the different anatomical location of measured alpha rhythms, the specific type of pain, or the presence of MDD comorbidity. It is possible that MDD patients with higher PAF might have higher acute pain sensitivity prior to developing chronic pain, and that these patients may have a different intrinsic structure or function of brain circuits that conveys a higher likelihood of developing comorbid chronic pain. The present results suggest the possibility of neural circuitry common to both chronic pain and depression, and that certain neurophysiological features may define a biotype of depression that is both more susceptible to developing comorbid pain and more responsive to rTMS treatment.

These findings also suggest that EEG biomarkers may help guide the search for an alternative rTMS approach for the treatment of MDD and pain comorbidity. We have identified a network including midline and sensorimotor regions that displays lower baseline phase-coherence for rTMS non-responders/non-remitters compared to responders/remitters among all patients with MDD and chronic pain comorbidity. This observation is consistent with the idea of network reorganization with chronic pain (Farmer, Baliki, & Apkarian, 2012). The topography of network should be further examined for its possible alignment with the dynamic pain connectome including the default mode network and/or salience network in chronic pain using source localization (Alshelh et al., 2018; Kucyi & Davis, 2017; Van Ettinger-Veenstra et al., 2019). Future studies also should examine whether the PAF or phase-coherence metrics in comorbid patients normalize with successful rTMS treatment.

Two testable hypotheses for future studies are that: (a) a multi-target rTMS approach combining the established left DLPFC target for depression with a secondary pain target within the sensorimotor-midline network will enhance antidepressant response to rTMS, and (b) successful rTMS treatment for comorbid MDD and pain would re-establish non-comorbid levels of PAF and sensorimotor-midline functional connectivity. While the EEG findings among different pain types are beyond the scope of the present data, differences in PAF and network synchrony may be specific to certain chronic pain conditions and such possible differences should be systematically examined in future studies.

### Limitations

The reported findings should be interpreted in the context of several limitations. First, these subjects were treated in a naturalistic setting with all individuals continuing their psychotropic medication during the rTMS course. Additionally, while all patients started with 10 Hz left DLPFC treatment, their stimulation protocol could be adjusted during the 30-session course according to a measurement-based care paradigm. While we did not detect a significant effect of medication or TMS parameters on outcome, it is possible that there may have been an interaction among types of concurrent medications and the rTMS effect on pain symptoms. Future trials should explicitly control for or exclude confounding medications. Second, we evaluated the severity of pain regardless of the specific etiology. Patients with fibromyalgia, neuropathic pain, chronic lower back pain, and complex regional pain syndrome were combined in this sample, despite their possibly different pathophysiological mechanisms. While rTMS has been previously shown to successfully improve various pain types, it remains unclear if the presence or direction of the association between pain symptoms and PAF or phase-coherence is clinically meaningful across different pain conditions. Follow-up research should systematically compare the effect of rTMS on different pain types.

## Conclusions

We present preliminary evidence that MDD comorbidity with chronic pain results in lower rTMS response/remission rates for depression and propose that increased PAF and hypoconnectivity between sensorimotor and midline regions may predict this worse rTMS therapeutic prognosis in the comorbid population. Understanding the underlying circuitry and developing a more targeted treatment approach may help further develop a therapy for this common comorbidity. A multi-target rTMS approach represents a promising avenue for non-pharmacological treatment for comorbid MDD and chronic pain.

## References

[ref1] Ahn, S., Prim, J. H., Alexander, M. L., McCulloch, K. L., & Fröhlich, F. (2019). Identifying and engaging neuronal oscillations by transcranial alternating current stimulation in patients with chronic low back pain: A randomized, crossover, double-blind, sham-controlled pilot study. Journal of Pain, 20(3), 277.e1–277.e11. doi: 10.1016/j.jpain.2018.09.004. PMC6382517 30268803

[ref2] Alshelh, Z., Marciszewski, K. K., Akhter, R., Di Pietro, F., Mills, E. P., Vickers, E. R., … Henderson, L. A. (2018). Disruption of default mode network dynamics in acute and chronic pain states. NeuroImage: Clinical, 17, 222–231. doi: 10.1016/j.nicl.2017.10.019. 29159039 PMC5683191

[ref3] Altas, E. U., Askin, A., Beşiroğlu, L., & Tosun, A. (2019). Is high-frequency repetitive transcranial magnetic stimulation of the left primary motor cortex superior to the stimulation of the left dorsolateral prefrontal cortex in fibromyalgia syndrome? Somatosensory & Motor Research, 36(1), 56–62. doi: 10.1080/08990220.2019.1587400. 30955403

[ref4] Arendsen, L. J., Hugh-Jones, S., & Lloyd, D. M. (2018). Transcranial alternating current stimulation at alpha frequency reduces pain when the intensity of pain is uncertain. Journal of Pain, 19(7), 807–818. doi: 10.1016/j.jpain.2018.02.014. 29551661

[ref5] Arns, M., Drinkenburg, W. H., Fitzgerald, P. B., & Kenemans, J. L. (2012). Neurophysiological predictors of non-response to rTMS in depression. Brain Stimulation, 5(4), 569–576. doi: 10.1016/j.brs.2011.12.003. 22410477

[ref6] Babiloni, C., Brancucci, A., Del Percio, C., Capotosto, P., Arendt-Nielsen, L., Chen, A. C. N., & Rossini, P. M. (2006). Anticipatory electroencephalography alpha rhythm predicts subjective perception of pain intensity. Journal of Pain, 7(10), 709–717. doi: 10.1016/j.jpain.2006.03.005. 17018331

[ref7] Bair, M. J., Robinson, R. L., Eckert, G. J., Stang, P. E., Croghan, T. W., & Kroenke, K. (2004). Impact of pain on depression treatment response in primary care. Psychosomatic Medicine, 66(1), 17–22. doi: 10.1097/01.PSY.0000106883.94059.C5. 14747633

[ref8] Bair, M. J., Robinson, R. L., Katon, W., & Kroenke, K. (2003). Depression and pain comorbidity: A literature review. Archives of Internal Medicine, 163(20), 2433–2445. doi: 10.1001/archinte.163.20.2433. 14609780

[ref9] Beam, W., Borckardt, J. J., Reeves, S. T., & George, M. S. (2009). An efficient and accurate new method for locating the F3 position for prefrontal TMS applications. Brain Stimulation, 2(1), 50–54. doi: 10.1016/j.brs.2008.09.006. 20539835 PMC2882797

[ref10] Cahill, C. M., & Taylor, A. M. (2017). Neuroinflammation – a co-occurring phenomenon linking chronic pain and opioid dependence. Current Opinion in Behavioral Sciences, 13, 171–177. doi: 10.1016/j.cobeha.2016.12.003. 28451629 PMC5404697

[ref11] Carpenter, L. L., Conelea, C., Tyrka, A. R., Welch, E. S., Greenberg, B. D., Price, L. H., … Philip, N. S. (2018). 5Hz Repetitive transcranial magnetic stimulation for posttraumatic stress disorder comorbid with major depressive disorder. Journal of Affective Disorders, 235, 414–420. doi: 10.1016/j.jad.2018.04.009. 29677606 PMC6567988

[ref12] Chang, M. H., Hsu, J. W., Huang, K. L., Su, T. P., Bai, Y. M., Li, C. T., … Chen, M. H. (2015). Bidirectional association between depression and fibromyalgia syndrome: A nationwide longitudinal study. Journal of Pain, 16(9), 895–902. doi: 10.1016/j.jpain.2015.06.004. 26117813

[ref13] Chiarotto, A., Maxwell, L. J., Ostelo, R. W., Boers, M., Tugwell, P., & Terwee, C. B. (2019). Measurement properties of visual analogue scale, numeric rating scale, and pain severity subscale of the brief pain inventory in patients with low back pain: A systematic review. Journal of Pain, 20, 245–263. doi: 10.1016/j.jpain.2018.07.009. 30099210

[ref14] Corlier, J., Carpenter, L. L., Wilson, A. C., Tirrell, E., Gobin, A. P., Kavanaugh, B., & Leuchter, A. F. (2019a). The relationship between individual alpha peak frequency and clinical outcome with repetitive transcranial magnetic stimulation (rTMS) treatment of major depressive disorder (MDD). Brain Stimulation, 12(6), 1572–1578. doi: 10.1016/j.brs.2019.07.018. 31378603

[ref15] Corlier, J., Wilson, A., Hunter, A. M., Vince-Cruz, N., Krantz, D., Levitt, J., … Leuchter, A. F. (2019b). Changes in functional connectivity predict outcome of repetitive transcranial magnetic stimulation treatment of major depressive disorder. Cerebral Cortex, 29(12), 4958–4967. doi: 10.1093/cercor/bhz035. 30953441 PMC7305800

[ref16] de Vries, M., Wilder-Smith, O. H. G., Jongsma, M. L. A., van den Broeke, E. N., Arns, M., van Goor, H., & van Rijn, C. M. (2013). Altered resting state EEG in chronic pancreatitis patients: Toward a marker for chronic pain. Journal of Pain Research, 6, 815–824. doi: 10.2147/JPR.S50919. 24379694 PMC3843642

[ref17] Farmer, M. A., Baliki, M. N., & Apkarian, A. V. (2012). A dynamic network perspective of chronic pain. Neuroscience Letters, 520, 197–203. doi: 10.1016/j.neulet.2012.05.001. 22579823 PMC3377811

[ref18] Fitzgerald, P. B., Hoy, K. E., Anderson, R. J., & Daskalakis, Z. J. (2016). A study of the pattern of response to rTMS treatment in depression. Depression and Anxiety, 33(8), 746–753. doi: 10.1002/da.22503. 27059158

[ref19] Fox, M. D., Halko, M. A., Eldaief, M. C., & Pascual-Leone, A. (2012). Measuring and manipulating brain connectivity with resting state functional connectivity magnetic resonance imaging (fcMRI) and transcranial magnetic stimulation (TMS). Human Brain Mapping Journal, 62(4), 2232–2243. 10.1016/j.neuroimage.2012.03.035 PMC3518426 22465297

[ref20] Furman, A. J., Meeker, T. J., Rietschel, J. C., Yoo, S., Muthulingam, J., Prokhorenko, M., … Seminowicz, D. A. (2018). Cerebral peak alpha frequency predicts individual differences in pain sensitivity. NeuroImage, 167, 203–210. doi: 10.1016/j.neuroimage.2017.11.042. 29175204

[ref21] Furman, A. J., Prokhorenko, M., Keaser, M. L., Zhang, J., Chen, S., Mazaheri, A., & Seminowicz, D. A. (2020). Sensorimotor peak alpha frequency is a reliable biomarker of prolonged pain sensitivity. Cerebral Cortex, 30(12), 6069–6082. doi: 10.1093/cercor/bhaa124. 32591813 PMC7732034

[ref22] Furman, A. J., Thapa, T., Summers, S. J., Cavaleri, R., Fogarty, J. S., Steiner, G. Z., … Seminowicz, D. A. (2019). Cerebral peak alpha frequency reflects average pain severity in a human model of sustained, musculoskeletal pain. Journal of Neurophysiology, 122(4), 1784–1793. doi: 10.1152/jn.00279.2019. 31389754 PMC6843105

[ref23] Galhardoni, R., Correia, G. S., Araujo, H., Yeng, L. T., Fernandes, D. T., Kaziyama, H. H., … de Andrade, D. C. (2015). Repetitive transcranial magnetic stimulation in chronic pain: A review of the literature. Archives of Physical Medicine and Rehabilitation, 96(4), S156–S172. doi: 10.1016/j.apmr.2014.11.010. 25437106

[ref24] George, M. S., Lisanby, S. H., Avery, D., McDonald, W. M., Durkalski, V., Pavlicova, M., … Sackeim, H. A. (2010). Daily left prefrontal transcranial magnetic stimulation therapy for major depressive disorder: A sham-controlled randomized trial. Archives of General Psychiatry, 67(5), 507–516. doi: 10.1001/archgenpsychiatry.2010.46. 20439832

[ref25] Gerrits, M. M. J. G., Van Oppen, P., Van Marwijk, H. W. J., Penninx, B. W. J. H., & Van Der Horst, H. E. (2014). Pain and the onset of depressive and anxiety disorders. Pain, 155(1), 53–59. doi: 10.1016/j.pain.2013.09.005. 24012953

[ref26] Gerrits, M. M. J. G., Vogelzangs, N., Van Oppen, P., Van Marwijk, H. W. J., Van Der Horst, H., & Penninx, B. W. J. H. (2012). Impact of pain on the course of depressive and anxiety disorders. Pain, 153(2), 429–436. doi: 10.1016/j.pain.2011.11.001. 22154919

[ref27] Goudra, B., Shah, D., Balu, G., Gouda, G., Balu, A., Borle, A., & Singh, P. (2017). Repetitive transcranial magnetic stimulation in chronic pain: A meta-analysis. Anesthesia: Essays and Researches, 11(3), 751. doi: 10.4103/aer.AER_10_17. 28928582 PMC5594801

[ref28] Gracely, R. H., Ceko, M., & Bushnell, M. C. (2012). Fibromyalgia and depression. Pain Research and Treatment, 2012. doi: 10.1155/2012/486590. PMC3236322 22191023

[ref29] Grandy, T. H., Werkle-Bergner, M., Chicherio, C., Schmiedek, F., Lövdén, M., & Lindenberger, U. (2013). Peak individual alpha frequency qualifies as a stable neurophysiological trait marker in healthy younger and older adults. Psychophysiology, 50(6), 570–582. doi: 10.1111/psyp.12043. 23551082

[ref30] Hamilton, M. (1960). A rating scale for depression. Journal of Neurology, Neurosurgery and Psychiatry, 23(1), 56–62. doi: 10.1136/jnnp.23.1.56. 14399272 PMC495331

[ref31] Heath, A., Taylor, J., & McNerney, M. W. (2018). rTMS for the treatment of Alzheimer's disease: Where should we be stimulating? Expert Review of Neurotherapeutics, 18(12), 903–905. doi: 10.1080/14737175.2018.1538792. 30350733 PMC6483370

[ref32] Hou, W.-H., Wang, T.-Y., & Kang, J.-H. (2016). The effects of add-on non-invasive brain stimulation in fibromyalgia: A meta-analysis and meta-regression of randomized controlled trials. Rheumatology, 55(8), 1507–1517. doi: 10.1093/rheumatology/kew205. 27150193

[ref33] Hsu, J. H., Daskalakis, Z. J., & Blumberger, D. M. (2018). An update on repetitive transcranial magnetic stimulation for the treatment of co-morbid pain and depressive symptoms. Current Pain and Headache Reports, 22(7), 51. doi: 10.1007/s11916-018-0703-7. 29904802

[ref34] Janicak, P. G., Dunner, D. L., Aaronson, S. T., Carpenter, L. L., Boyadjis, T. A., Brock, D. G., … Demitrack, M. A. (2013). Transcranial magnetic stimulation (TMS) for major depression: A multisite, naturalistic, observational study of quality of life outcome measures in clinical practice. CNS Spectrums, 18(6), 322–332. doi: 10.1017/S1092852913000357. 23895940

[ref35] Johnson, S., Summers, J., & Pridmore, S. (2006). Changes to somatosensory detection and pain thresholds following high frequency repetitive TMS of the motor cortex in individuals suffering from chronic pain. Pain, 123(1–2), 187–192. doi: 10.1016/j.pain.2006.02.030. 16616419

[ref36] Karcioglu, O., Topacoglu, H., Dikme, O., & Dikme, O. (2018). A systematic review of the pain scales in adults: Which to use? American Journal of Emergency Medicine, 36, 707–714. doi: 10.1016/j.ajem.2018.01.008. 29321111

[ref37] Kim, J. A., Bosma, R. L., Hemington, K. S., Rogachov, A., Osborne, N. R., Cheng, J. C., … Davis, K. D. (2019). Neuropathic pain and pain interference are linked to alpha-band slowing and reduced beta-band magnetoencephalography activity within the dynamic pain connectome in patients with multiple sclerosis. Pain, 160(1), 187–197. doi: 10.1097/j.pain.0000000000001391. 30188456

[ref38] Kim, J. A., & Davis, K. D. (2020). Neural oscillations: Understanding a neural code of pain. Neuroscientist, 1073858420958629. doi: 10.1177/1073858420958629. 32981457

[ref39] Kisler, L. B., Kim, J. A., Hemington, K. S., Rogachov, A., Cheng, J. C., Bosma, R. L., … Davis, K. D. (2020). Abnormal alpha band power in the dynamic pain connectome is a marker of chronic pain with a neuropathic component. NeuroImage: Clinical, 26. doi: 10.1016/j.nicl.2020.102241 PMC7090370 32203904

[ref40] Knijnik, L. M., Dussán-Sarria, J. A., Rozisky, J. R., Torres, I. L. S., Brunoni, A. R., Fregni, F., & Caumo, W. (2016). Repetitive transcranial magnetic stimulation for fibromyalgia: Systematic review and meta-analysis. Pain Practice, 16(3), 294–304. doi: 10.1111/papr.12276. 25581213

[ref41] Krepel, N., Rush, A. J., Iseger, T. A., Sack, A. T., & Arns, M. (2019). Can psychological features predict antidepressant response to rTMS? A discovery-replication approach. Psychological Medicine, 50(2), 264–272. doi: 10.1017/S0033291718004191. 30674359

[ref42] Kucyi, A., & Davis, K. D. (2017). The neural code for pain: From single-cell electrophysiology to the dynamic pain connectome. Neuroscientist, 23, 397–414. doi: 10.1177/1073858416667716. 27660241

[ref43] Lee, J. C., Wilson, A. C., Corlier, J., Tadayonnejad, R., Marder, K. G., Pleman, C. M., … Leuchter, A. F. (2020). Strategies for augmentation of high-frequency left-sided repetitive transcranial magnetic stimulation treatment of major depressive disorder. Journal of Affective Disorders, 277, 964–969. doi: 10.1016/j.jad.2020.09.011. 33065840

[ref44] Lefaucheur, J. P., Aleman, A., Baeken, C., Benninger, D. H., Brunelin, J., Di Lazzaro, V., … Ziemann, U. (2020). Evidence-based guidelines on the therapeutic use of repetitive transcranial magnetic stimulation (rTMS): An update (2014–2018). Clinical Neurophysiology, 131, 474–528. doi: 10.1016/j.clinph.2019.11.002. 31901449

[ref45] Lefaucheur, J. P., André-Obadia, N., Antal, A., Ayache, S. S., Baeken, C., Benninger, D. H., … Garcia-Larrea, L. (2014). Evidence-based guidelines on the therapeutic use of repetitive transcranial magnetic stimulation (rTMS). Clinical Neurophysiology, 125, 2150–2206. doi: 10.1016/j.clinph.2014.05.021. 25034472

[ref46] Leuchter, A. F., Hunter, A. M., Krantz, D. E., & Cook, I. A. (2015). Rhythms and blues: Modulation of oscillatory synchrony and the mechanism of action of antidepressant treatments. Annals of the New York Academy of Sciences, 1344(1), 78–91. doi: 10.1111/nyas.12742. 25809789 PMC4412810

[ref47] Leuchter, A. F., Husain, M. M., Cook, I. A., Trivedi, M. H., Wisniewski, S. R., Gilmer, W. S., … Rush, A. J. (2010). Painful physical symptoms and treatment outcome in major depressive disorder: A STAR*D (Sequenced Treatment Alternatives to Relieve Depression) report. Psychological Medicine, 40(2), 239–251. doi: 10.1017/S0033291709006035. 19493369

[ref48] Lopez-Diaz, K., Henshaw, J., Casson, A. J., Brown, C. A., Taylor, J. R., Trujillo-Barreto, N. J., … Sivan, M. (2021). Alpha entrainment drives pain relief using visual stimulation in a sample of chronic pain patients: A proof-of-concept controlled study. NeuroReport, 32(5), 394–398. doi: 10.1097/WNR.0000000000001606. 33661810

[ref49] Maletic, V., & Raison, C. L. (2009). Neurobiology of depression, fibromyalgia and neuropathic pain. Frontiers in Bioscience, 14(14), 5291–5338. doi: 10.2741/3598. 19482616

[ref50] Meneses, F. M., Queirós, F. C., Montoya, P., Miranda, J. G. V., Dubois-Mendes, S. M., Sá, K. N., … Baptista, A. F. (2016). Patients with rheumatoid arthritis and chronic pain display enhanced alpha power density at rest. Frontiers in Human Neuroscience, 10, 11. doi: 10.3389/fnhum.2016.00395. 27540360 PMC4972828

[ref51] Mori, F., Codecà, C., Kusayanagi, H., Monteleone, F., Buttari, F., Fiore, S., … Centonze, D. (2010). Effects of anodal transcranial direct current stimulation on chronic neuropathic pain in patients with multiple sclerosis. Journal of Pain, 11(5), 436–442. doi: 10.1016/j.jpain.2009.08.011. 20018567

[ref52] Nolan, H., Whelan, R., & Reilly, R. B. (2010). FASTER: Fully automated statistical thresholding for EEG artifact rejection. Journal of Neuroscience Methods, 192(1), 152–162. doi: 10.1016/j.jneumeth.2010.07.015. 20654646

[ref53] Onesti, E., Gabriele, M., Cambieri, C., Ceccanti, M., Raccah, R., Di Stefano, G., … Inghilleri, M. (2013). H-coil repetitive transcranial magnetic stimulation for pain relief in patients with diabetic neuropathy. European Journal of Pain, 17(9), 1347–1356. doi: 10.1002/j.1532-2149.2013.00320.x. 23629867

[ref54] Petrosino, N. J., Zandvakili, A., Carpenter, L. L., & Philip, N. S. (2018). Pilot testing of peak alpha frequency stability during repetitive transcranial magnetic stimulation. Frontiers in Psychiatry, 9, 605. doi: 10.3389/fpsyt.2018.00605. PMC6256033 30515110

[ref55] Phillips, A. L., Burr, R. L., & Dunner, D. L. (2018). rTMS effects in patients with co-morbid somatic pain and depressive mood disorders. Journal of Affective Disorders, 241, 411–416. doi: 10.1016/j.jad.2018.08.065. 30145511

[ref56] Ploner, M., Sorg, C., & Gross, J. (2017). Brain rhythms of pain. Trends in Cognitive Sciences, 21, 100–110. doi: 10.1016/j.tics.2016.12.001. 28025007 PMC5374269

[ref57] Saltychev, M., & Laimi, K. (2017). Effectiveness of repetitive transcranial magnetic stimulation in patients with fibromyalgia. International Journal of Rehabilitation Research, 40(1), 11–18. doi: 10.1097/MRR.0000000000000207. 27977465

[ref58] Seminowicz, D. A., Thapa, T., Furman, A. J., Summers, S. J., Cavaleri, R., Fogarty, J. S., … Schabrun, S. M. (2018). Slow peak alpha frequency and corticomotor depression linked to high pain susceptibility in transition to sustained pain. BioRxiv, 278598. doi:10.1101/278598.

[ref59] Sheehan, D. V., Lecrubier, Y., Sheehan, K. H., Amorim, P., Janavs, J., Weiller, E., … Dunbar, G. C. (1998). The Mini-International Neuropsychiatric Interview (M.I.N.I.): The development and validation of a structured diagnostic psychiatric interview for DSM-IV and ICD-10. Journal of Clinical Psychiatry, 59(SUPPL. 20), 22–33. 9881538

[ref60] Short, B. E., Borckardt, J. J., Anderson, B. S., Frohman, H., Beam, W., Reeves, S. T., & George, M. S. (2011). Ten sessions of adjunctive left prefrontal rTMS significantly reduces fibromyalgia pain: A randomized, controlled pilot study. Pain, 152(11), 2477–2484. doi: 10.1016/j.pain.2011.05.033. 21764215 PMC3199360

[ref61] Sinyor, M., Schaffer, A., & Levitt, A. (2010). The sequenced treatment alternatives to relieve depression (STAR*D) trial: A review. Canadian Journal of Psychiatry, 55, 126–135. doi: 10.1177/070674371005500303. 20370962

[ref62] Soleimani, R., Jalali, M. M., & Hasandokht, T. (2016). Therapeutic impact of repetitive transcranial magnetic stimulation (rTMS) on tinnitus: A systematic review and meta-analysis. European Archives of Oto-Rhino-Laryngology, 273, 1663–1675. doi: 10.1007/s00405-015-3642-5. 25968009

[ref63] Sullivan, M. D., Edlund, M. J., Steffick, D., & Unützer, J. (2005). Regular use of prescribed opioids: Association with common psychiatric disorders. Pain, 119(1–3), 95–103. doi: 10.1016/j.pain.2005.09.020. 16298066

[ref64] Tadayonnejad, R., Wilson, A. C., Corlier, J., Lee, J. C., Ginder, N. D., Levitt, J. G., … Leuchter, A. F. (2020). Sequential multi-locus transcranial magnetic stimulation for treatment of obsessive-compulsive disorder with comorbid major depression: A case series. Brain Stimulation, 13, 1600–1602. doi: 10.1016/j.brs.2020.10.003. 33065361

[ref65] Taylor, A. M. W., Becker, S., Schweinhardt, P., & Cahill, C. (2016). Mesolimbic dopamine signaling in acute and chronic pain: Implications for motivation, analgesia, and addiction. Pain, 157(6), 1194–1198. doi: 10.1097/j.pain.0000000000000494. 26797678 PMC4866581

[ref66] To, W. T., De Ridder, D., Hart Jr., J., & Vanneste, S. (2018). Changing brain networks through non-invasive neuromodulation. Frontiers in Human Neuroscience, 12(April), 1–17. 10.3389/fnhum.2018.00128. 29706876 PMC5908883

[ref67] Trivedi, M. H., Rush, A. J., Ibrahim, H. M., Carmody, T. J., Biggs, M. M., Suppes, T., … Kashner, T. M. (2004). The inventory of depressive symptomatology, clinician rating (IDS-C) and self-report (IDS-SR), and the quick inventory of depressive symptomatology, clinician rating (QIDS-C) and self-report (QIDS-SR) in public sector patients with mood disorders: A psychometric evaluation. Psychological Medicine, 34(1), 73–82. 14971628 10.1017/s0033291703001107

[ref68] Van Ettinger-Veenstra, H., Lundberg, P., Alföldi, P., Södermark, M., Graven-Nielsen, T., Sjörs, A., … Gerdle, B. (2019). Chronic widespread pain patients show disrupted cortical connectivity in default mode and salience networks, modulated by pain sensitivity. Journal of Pain Research, 12, 1743–1755. doi: 10.2147/JPR.S189443. 31213886 PMC6549756

[ref69] Vinck, M., Oostenveld, R., Van Wingerden, M., Battaglia, F., & Pennartz, C. M. A. (2011). An improved index of phase-synchronization for electrophysiological data in the presence of volume-conduction, noise and sample-size bias. NeuroImage, 55(4), 1548–1565. doi: 10.1016/j.neuroimage.2011.01.055. 21276857

[ref70] Von Korff, M., & Simon, G. (1996). The relationship between pain and depression. British Journal of Psychiatry, 168(JUNE SUPP. 30), 101–108. doi: 10.1192/s0007125000298474. 8864155

[ref71] Walker, A. K., Kavelaars, A., Heijnen, C. J., & Dantzer, R. (2014). Neuroinflammation and comorbidity of pain and depression. Pharmacological Reviews, 66(1), 80–101. doi: 10.1124/pr.113.008144. 24335193 PMC3880465

[ref72] Wang, W. E., Roy, A., Misra, G., Ho, R. L. M., Ribeiro-Dasilva, M. C., Fillingim, R. B., … Coombes, S. A. (2019). Altered neural oscillations within and between sensorimotor cortex and parietal cortex in chronic jaw pain. NeuroImage: Clinical, 24, 101964. doi: 10.1016/j.nicl.2019.101964. PMC6704052 31412309

[ref73] Xing, M., Ajilore, O., Wolfson, O. E., Abbott, C., Macnamara, A., Tadayonnejad, R., … Leow, A. (2016a). Thought chart: Tracking dynamic EEG brain connectivity with unsupervised manifold learning. Lecture Notes in Computer Science (Including Subseries Lecture Notes in Artificial Intelligence and Lecture Notes in Bioinformatics), 9919 LNAI, 149–157. doi:10.1007/978-3-319-47103-7_15.

[ref74] Xing, M., Tadayonnejad, R., Macnamara, A., Ajilore, O., Phan, K. L., Klumpp, H., & Leow, A. (2016b). EEG based functional connectivity reflects cognitive load during emotion regulation. Proceedings – International Symposium on Biomedical Imaging, 2016-June, 771–774. doi:10.1109/ISBI.2016.7493380.

